# Publication Trends and Their Relationship With Academic Success Among Dermatology Residents: Cross-sectional Analysis

**DOI:** 10.2196/30015

**Published:** 2021-10-06

**Authors:** J Michael Anderson, David Wenger, Austin L Johnson, Corbin Walters, Mopileola Tomi Adewumi, Lindy Esmond, Jourdan Waddell, Matt Vassar

**Affiliations:** 1 Oklahoma State University Center for Health Sciences Tulsa, OK United States

**Keywords:** publication trends, dermatology residency, academic medicine

## Abstract

**Background:**

Involvement in scholarly activities is considered to be one of the foundational pillars of medical education.

**Objective:**

This study aims to investigate publication rates before, during, and after residency to determine whether research productivity throughout medical training correlates with future academic success and research involvement.

**Methods:**

We successfully identified a list of 296 graduates from 25 US dermatology residency programs from the years 2013-2015. The publication history for each graduate was compiled using Scopus, PubMed, and Google Scholar. The Pearson correlation test and linear regression were used to assess the relationship between research productivity and continued academic success after residency graduation.

**Results:**

Before residency, graduates published a mean of 1.9 (SD 3.5) total publications and a mean of 0.88 (SD 1.5) first-author publications. During residency, graduates published a mean of 2.7 (SD 3.6) total publications and a mean of 1.39 (SD 2.0) first-author publications. Graduates who pursued a fellowship had more total publications (*t*_294_=−4.0; *P*<.001), more first-author publications (*t*_294_=−3.9; *P*<.001), and a higher h-index (*t*_294_=−3.8; *P*=.002). Graduates who chose to pursue careers in academic medicine had more mean total publications (*t*_294_=−7.5; *P*<.001), more first-author publications (*t*_294_=−5.9; *P*<.001), and a higher mean h-index (*t*_294_=−6.9; *P*<.001). Graduates with one or more first-author publications before residency were 1.3 times more likely to pursue a career in academic medicine (adjusted odds ratio 1.3, 95% CI 1.1-1.5). Graduates who pursued a fellowship were also 1.9 times more likely to pursue a career in academic medicine (adjusted odds ratio 1.9, 95% CI 1.2-3.2).

**Conclusions:**

Our results suggest that research productivity before and during residency training are potential markers for continued academic success and research involvement after completing dermatology residency training.

## Introduction

### Background

Successful matching into selective residency programs, such as dermatology, is multifactorial in nature and requires thoughtful planning by medical students to ensure that they have a competitive, well-rounded application. Previous studies suggest that higher medical licensing exam scores (eg, United States Medical Licensing Exam [USMLE] step 1 and step 2 clinical knowledge scores), honor society memberships, and medical school rankings are associated with an increased likelihood of successfully matching into a residency program [1,2]. Beyond these objective measures, an applicant's research experiences—in the form of abstracts, presentations, and peer-reviewed publications—are an important component in the residency application process [3]. A 2011 survey of medical school graduates who successfully matched into a dermatology residency program found that >85% of graduates listed publications on their Electronic Residency Application Service application. In this cohort of graduates, the average number of publications before matching was >5 total publications per graduate. As the importance placed on early research exposure has increased, more medical students may elect to participate in research during medical school to enhance their residency application, given that research is a core requirement placed on residency programs and program coordinators to maintain the program's accreditation status [4].

Since its conception in 1994, the Accreditation Council for Graduate Medical Education (ACGME) [5] has required research participation by residency programs and their residents during training. These requirements mandate that residency programs educate residents on the “basic principles of scientific inquiry, including how research is designed, conducted, evaluated, explained to patients, and applied to patient care” [6] and that residents must then engage in scholarly activities as part of their training. Despite mandating these scholarly requirements for accreditation, previous studies have shown that residency programs often fall short of meeting such requirements [7]. Although efforts have been made to determine the level of research participation by residents in other medical specialties [8-10], little is known regarding the extent to which dermatology residents participate in scholarly activities.

Here, we sought to identify whether a correlation exists between research productivity of dermatology residency graduates with continued academic successes and research involvement (eg, careers in academic medicine vs private practice) or whether those with higher research output elected to pursue a fellowship upon completion of residency training. Thus, we explore current research practices and publication trends of dermatology residency graduates to determine whether research efforts made during medical training are associated with future academic achievements (in the form of peer-reviewed publications). Furthermore, we assess whether higher research output during residency correlated with the pursuit of fellowship training or a career in academic medicine.

### Objectives

Our primary objectives are to (1) quantify the number of peer-reviewed publications per resident during the periods before, during, and after residency training and (2) determine whether increased research productivity and academic success (eg, number of peer-reviewed publications and individual h-index scores) are associated with future academic production (eg, careers in academic medicine vs private practice).

## Methods

### Residency Program Selection

We used the Doximity Residency Navigator to generate our sample of dermatology residency programs. The Doximity residency ranking is based on subjective reviews of programs that combine objective data, such as alumni research output and board examination pass rate, with subjective data, including current and graduate resident satisfaction scores and *reputation data*, which is collected from past and present residents on an annual basis [11].

To identify top US dermatology residency programs and graduates, we used a search strategy similar to that performed by Yang et al [8]. This process entailed one of the authors (JMA) searching the 2019-2020 Doximity Residency Navigator using the *Dermatology* specialty search tool. Next, the programs were sorted as A-Z and exported to a Microsoft Excel document. Finally, we assigned a random number to each residency program using Microsoft Excel’s random number generator.

After randomization, we selected the first 50 residency programs to be included in our sample. Next, we searched for the names of residency graduates (graduating in the years 2013, 2014, and 2015) on each program’s institutional website. If this search was unsuccessful, we searched for the name and email address of each residency coordinator using the advanced program search on the ACGME website [12]. We attempted to retrieve a list of residency graduates from each program coordinator. This email correspondence, which has been used in previous studies [13,14], was included to increase the cogency of our methodology. Furthermore, we used the same standardized email process, which entailed repeating the attempted email correspondence one time per week for 3 consecutive weeks, as used in a systematic review by Song et al [15]. Finally, we allotted program coordinators 8 weeks from the date of the initial email to respond before deeming that program *noncontactable*. If no response was received or if the email was returned as *inactive*, the program was excluded, and a subsequent program was randomly selected from the original list of residency programs, and the above process was repeated until a 50% inclusion rate was met.

### Training

To ensure consistency among investigators, 3 of the authors (DW, LE, and JW) completed in-person training before data extraction. During this training session, the following items were addressed and discussed at length: (1) description of study design and objectives, (2) a thorough review of the study protocol, (3) step-by-step instructions on how to use the standardized Google form for extraction, and (4) discussion of specific data points to be extracted. The Google form was pilot-tested by each investigator during training with the help of 3 residency graduates and their publication history as examples. After pilot testing, data were extracted for the next 10 graduates in our sample. Responses were subsequently discussed, and any discrepancies among investigators were resolved before proceeding to the remaining list of graduates.

### Screening and Data Extraction

**Figure 1:**
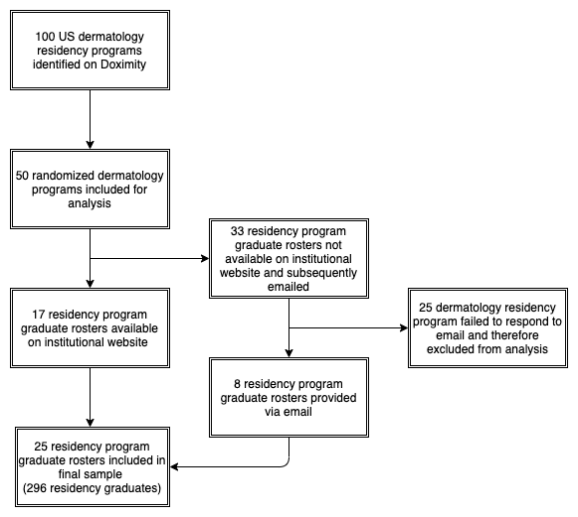
Stepwise approach used to identify peer-reviewed publications for dermatology residency graduates.

After training, 3 of the authors (DW, LE, and JW) extracted data in triplicate, independent, and blinded fashion. Extraction began on October 5, 2019, and concluded on September 10, 2020. To obtain a comprehensive publication history, we searched for each graduate on Scopus using the following demographic information: (1) full name, (2) institution, (3) residency program, (4) fellowship program, and (5) area of interest (dermatology). The list of publications returned for each graduate using this information was subsequently compared with the list of publications generated by searching for authors (using the same demographic information as above) on PubMed and Google Scholar searches. Results from the three individual searches were compared to ensure an accurate record of total publications per resident. More specifically, a publication was included only if it was included in the search return for all three databases. After we compiled a list of publications for each graduate, we extracted the following information from each publication: (1) type of publication, (2) year of publication, and (3) graduate affiliation at the time of publication. In addition, the author h-index was recorded (Figure 1).

### Data Analysis

Data were separated into the following three cohorts of time: before residency (including undergraduate and medical school education), during residency (4 years in duration in the United States), and after residency (including fellowship training, if applicable). The decision was made to include a 6-month overlap period to capture publications that were likely initiated and completed during the previous period. For example, publications that were published in the first 6 months of residency were classified as *before residency* as these studies were likely started during the *before residency* time frame, given the length of time required to conduct a research project, complete the peer review process, and see a research question through to publication. The results were presented as frequencies and percentages. We used a Pearson product coefficient to examine the relationships among each publication time frame (before, during, and after residency). An independent sample two-tailed *t* test was used to compare the mean number of publications for graduates who elected to enter academic medicine with those who entered private practice after completing their residency training. We also used an independent sample two-tailed *t* test to compare the mean number of publications between those who pursued fellowship training with those who did not. Binary logistic regression was used to analyze the relationship between career type (academic or private practice) and total author publications and fellowships, controlling for gender. Analyses were performed using STATA 15.1 (StataCorp, LLC).

## Results

### Overview

**Figure 2:**
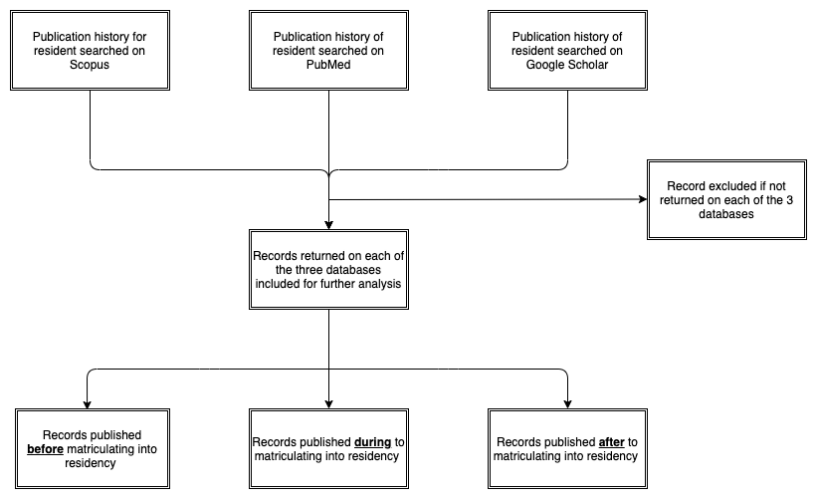
Program and resident inclusion flowchart.

A total of 100 US dermatology programs were found on the Doximity website. Of the 50 randomly sampled programs, we were able to locate a list of graduates for 17 (34%) programs using institutional websites. For the remaining 66% (33/50) programs, we attempted to obtain this list via email from each program coordinator. An additional 24% (8/33) programs provided a complete list of residency graduates via email correspondence. The remaining 76% (25/33) programs did not respond by the end of the 8-week time frame. Of the 50 sampled programs, 25 (50%) dermatology residency programs were included in total (Figure 2).

### Subject and Publication Characteristics

A total of 296 graduates were included in our final sample. Most graduates were female (222/296, 75%). Approximately 35.5% (105/296) graduates pursued a fellowship, with the most common being Mohs surgery (27/105, 25.7%), pediatric dermatology (20/105, 19%), dermatopathology (16/105, 15.2%), and procedural dermatology (15/105, 14.3%). Approximately 25% (74/296) of graduates entered academic medicine. Of the 105 graduates who pursued fellowship training, 45 (42.9%) also went on to pursue a career in academic medicine. The average h-index among all residency graduates was 3.6 (range 0-24; Table 1).

**Table 1:** Resident graduate sample characteristics (N=296).

Characteristics		Value, n (%, 95% CI)
**Sex**		
	Female	222 (75, 70.1 to 79.9)
	Male	74 (25, 20.1 to 29.9)
**Medical degree obtained**		
	MD^a^	295 (99.7, 99 to 100.3)
	DO^b^	1 (0.3, −0.3 to 0.9)
**Current setting of practice**		
	Private	222 (75, 70.1 to 79.9)
	Academic	74 (25, 20.1 to 29.9)
**Pursued fellowship**		
	No	191 (64.5, 59.1 to 70)
	Yes	105 (35.5, 30 to 40.9)
**Fellowships (n=105)**		
	Mohs surgery	27 (25.7, 17.4 to 34.1)
	Pediatric dermatology	20 (19, 11.5 to 26.6)
	Dermatopathology	16 (15.2, 8.4 to 22.1)
	Procedural dermatology	15 (14.3, 7.6 to 21)
	Clinical research	9 (8.6, 3.2 to 13.9)
	Cutaneous oncology or melanoma	7 (6.7, 1.9 to 11.4)
	Laser and aesthetic surgery	4 (3.8, 0.1 to 7.5)
	Cosmetic dermatology	3 (2.9, −0.3 to 6)
	Rheumatology	3 (2.9, −0.3 to 6)
	Biotechnology	1 (0.9, −0.9 to 2.8)
**h-index**		
	0	50 (16.9, 12.6 to 21.2)
	1-5	183 (61.8, 56.3 to 67.4)
	6-10	44 (14.9, 10.8 to 18.9)
	11-15	13 (4.4, 2.1 to 6.7)
	>15	6 (2, 0.4 to 3.6)
**Number of publications per resident**		
	0	39 (13.2, 9.3 to 17)
	1-5	129 (43.6, 37.9 to 49.2)
	6-10	53 (17.9, 13.5 to 22.3)
	11-15	27 (9.1, 5.8 to 12.4)
	16-20	15 (5.1, 2.6 to 7.6)
	21-25	12 (4.1, 1.8 to 6.3)
	26-30	8 (2.7, 0.8 to 4.6)
	>30	13 (4.4, 2.1 to 6.7)

^a^MD: doctor of medicine.

^b^DO: doctor of osteopathic medicine.

### Publications

Before residency, graduates had a mean of 1.9 (SD 3.5) total publications and a mean of 0.88 (SD 1.5) first-author publications. During residency, graduates had a mean of 2.7 (SD 3.6) total publications and a mean of 1.39 (SD 2.0) first-author publications (Table 2). Residents who graduated in 2013 produced a total of 889 (9.6 publications per person) publications, 2014 graduates produced 803 (7.44 per person) publications, and 2015 graduates produced 753 (7.93 per person) publications. A moderate positive correlation existed between the number of publications obtained before and during residency (*r*=0.35) and the number of publications obtained during residency and after residency training (*r*=0.37). A weak correlation was present between publications before residency and total publications after residency (*r*=0.19).

Graduates who pursued a fellowship had more total publications (*t*_294_=−4.0; *P*<.001), first-author publications (*t*_294_=−3.9; *P*<.001), and higher h-index (*t*_294_=−3.8; *P*=.002) than graduates who did not pursue fellowship training. In a similar manner, we found that graduates who chose to go into academic medicine had a higher number of mean total publications (*t*_294_=−7.5; *P*<.001), first-author publications (*t*_294_=−5.9; *P*<.001), and mean h-index (*t*_294_=−6.9; *P*<.001) than those going into private practice (Table 3).

**Table 2:** Mean and median publications per resident before, during, and after completion of residency training.

Author position			Value, mean (SD)		Value, median (IQR)		Total publications among all residents, n (%)
**Any author position**			8.3 (1.6)		5 (1-11)		2445 (100)
	Before	1.9 (0.40)		1 (0-2)		577 (23.59)	
	During	2.7 (0.54)		2 (0-4)		800 (32.72)	
	After	3.6 (1.02)		1 (0-4)		1068 (43.68)	
**First-author position**			3.14 (0.52)		0 (0-5)		965 (100)
	Before	0.9 (0.18)		0.9 (0-1)		261 (27.05)	
	During	1.4 (0.29)		1 (0-2)		411 (42.59)	
	After	1 (0.25)		0 (0-1)		293 (30.36)	

**Table 3:** Association between research productivity and pursuit of fellowship training, career in academic medicine, and gender (N=296).

		Total publications				Total first-author publications				h-index		
		Value, mean (SD)	*t* test (*df*)	*P* value	Value, mean (SD)		*t* test (*df*)	*P* value	Value, mean (SD)		*t* test (*df*)	*P* value
**All residency graduates**												
	Overall	8.3 (1.2)	N/A^a^	N/A^.^	1.2 (0.23)		N/A^.^	N/A	3.7 (0.45)		N/A^.^	N/A^.^
**Fellowship**												
	Yes	11.5 (2.3)	−4.0 (104)	<.001^b^	4.4 (0.35)		−3.9 (104)	<.001^b^	4.8 (0.79)		−3.8 (104)	.002^b^
	No	6.5 (1.3)	−4.0 (190)	<.001^b^	2.6 (0.29)		−3.9 (190)	<.001^b^	3 (0.53)		−3.8 (190)	.002^b^
**Career path**												
	Academic medicine	14.2 (1.7)	−7.5 (197)	<.001^b^	5 (0.48)		−5.9 (197)	<.001^b^	5.8 (0.99)		−6.9 (197)	<.001^b^
	Private practice	5.3 (0.9)	−7.5 (97)	<.001^b^	2.4 (0.24)		−5.9 (97)	<.001^b^	2.6 (0.40)		−6.9 (97)	<.001^b^
**Gender**												
	Male	10 (2.7)	−1.7 (73)	.09	3.9 (0.61)		−1.7 (73)	.09	3.9 (0.98)		−0.78 (73)	.44
	Female	7.7 (1.3)	−1.7 (221)	.09	3 (0.23)		−1.7 (221)	.09	3.5 (0.51)		−0.78 (221)	.44

^a^N/A: not applicable.

^b^Statistical significance was set at *P*<.005.

Our logistic regression model examined the relationship between first-author publications before residency and pursuit of fellowship training, as well as whether the graduate went into academic medicine. Graduates with one or more first-author publications were 1.3 times more likely to pursue a career in academic medicine than those with no first-author publications before residency (adjusted odds ratio 1.3, 95% CI 1.1-1.5). Graduates who pursued a fellowship were also 1.9 times more likely to enter into a career in academic medicine than those who did not pursue a fellowship (adjusted odds ratio 1.9, 95% CI 1.2-3.2).

## Discussion

### Principal Findings

Our results indicate that the total number of publications, first-author publications, and author h-index scores are highly associated with the pursuit of fellowship training, as well as entering into academic medicine following completion of dermatology residency training. Of the graduates included in our sample, over one-third elected to pursue a career in academic medicine, and one-third pursued fellowship training upon graduation. Residency graduates with at least one first-author publication before starting residency were more likely to pursue a career in academic medicine and continue their postgraduate education through fellowship subspecialty training. This emphasis on research appears to carry over into residency training, as we observed that the highest mean research output among the included dermatology graduates occurred during their years of residency training. Here, we discuss the implications that our findings may have on the dermatology match process for prospective applicants, as well as discuss how research throughout medical training may help open doors to future career opportunities and specialized fellowship training.

Our results demonstrate that dermatology residents published, on average, 2.7 (SD 0.54) articles during residency, with an average of 1.3 (SD 0.29) first-author publications. The research productivity among residents included in our sample is similar to that of residents in other fields [8,9,16]. These results are likely attributable to a recent push by the ACGME and individual residency locations to increase resident exposure to research activities [17,18]. Research involvement during residency promotes a well-rounded educational experience during residency—with a particular focus on evidence-based medicine—thereby strengthening resident confidence in research design and methodology, and it has been shown to be associated with higher clinical competency scores [19]. Stevenson et al [20] concluded that residency programs offering protected research time, established research curricula, and providing a specialized research track had increased residency scholarly activity, including the total number of publications. Perhaps integrating research into a program’s curriculum will not only ensure that the program is compliant with ACGME standards but also provide an opportunity for residents to establish a track record of scholarly successes. This increased research output during residency makes graduates more competitive for fellowship training positions, increases the likelihood of practicing in academia, and supports mentorship and networking opportunities [21].

Research productivity in the form of total publications, first-author publications, and higher author h-index scores was associated with the pursuit of fellowship training and academic medicine positions after completion of residency training. A recent study in the field of surgical oncology indicates that, along with research, factors such as attending a university-based residency, attending a residency associated with fellowship programs, and attending an allopathic medical school have an effect on matching into a fellowship [22]. Our results suggest that research during residency is associated with an increased likelihood of pursuing fellowship training in dermatology after completion of residency training. Although a higher total number of publications was observed among residents who pursued fellowship positions, previous research showing more career publications among residents who pursued additional training is conflicting. For example, Yang et al [8] found a strong association between the number of publications of urology residents during and after residency training. In contrast, Prasad et al [23] found that the number of total publications is a poor predictor of future publication among internal medicine residency graduates who pursue fellowship training. These contrasting findings may be the result of varying expectations of scholarly involvement among medical specialties. Despite the disconnect between early scholarly activity and continued research production among specialties, program directors (PDs) may still place emphasis on scholarly involvement when evaluating residency applicants.

Although PDs have many responsibilities, some of the key responsibilities include developing, overseeing, and improving their residency program’s education [24], as well as making crucial decisions in selecting residents who are most likely to be successful in their respective fields. In fields such as dermatology, where applicants outnumber available residency positions [25], PDs have historically relied on several metrics to stratify applicants. A major metric heavily considered by dermatology PDs for interview selection is the USMLE step 1 score [26]. Recently, the USMLE step 1 scoring reporting system changed from a 3-digit official score to a pass or fail system [27]. This modification of the step 1 scoring indicates that PDs will rely on other objective measures to stratify qualified applicants for interviews in the future. A potential stratification measure is research productivity in medical schools. For example, a recent survey of PDs suggests an increasing emphasis on research production as a potential stratification model for applicant selection [28]. Although previous studies have shown that other measures, including letters of recommendation, performance on audition clerkship rotations, and scholarships in medical school, are associated with success in residency training [29], the skills involved in research production are an underpinning of traits associated with good clinical practice. For example, participation in research has been shown to increase ethical awareness [30], teamwork and communication skills [31], and the ability to critically evaluate and synthesize new evidence [32], all of which are essential to becoming a competent physician.

In the 2018 match, dermatology yielded one of the lowest match percentages, with only 81.6% of applicants successfully matching, second only to interventional radiology [25]. Osteopathic and international medical graduates have historically low rates of matching competitive specialties, such as dermatology [33,34]. A common strategy taken by medical students, especially osteopathic and international medical graduates medical students who have lower match rates in dermatology programs compared with their allopathic counterparts, is to complete an extra research year between graduating medical school and applying for residency positions to increase their competitiveness. As higher research productivity during preclinical training years has been shown to increase the chances of successfully matching into a dermatology program [35], research remains one of the modifiable factors for prospective residency applicants. Of note, it is important for PDs to take into account potential limitations to research resources available to each applicant depending on their background or school attended. As an alternative to considering peer-reviewed publications as the sole measure of research success, we contend that PDs should also place emphasis on applicants’ enthusiasm and desire to participate in research. For instance, applicants may seek out opportunities that may have not resulted in a peer-reviewed publication but still provided the opportunity to develop a research question, conceptualize and implement a study protocol, and demonstrate the ability to think critically while attempting to answer critical research questions.

Our study has both strengths and limitations. In regard to the limitations, a metric used for comparing research production was the author h-index. Although the h-index is considered a robust metric, it does not account for authorship order, which may limit our ability to determine the extent of an author’s involvement in the associated research projects [36]. In addition, the cross-sectional nature of our study prevents the generalization of our results to other periods or fields of medicine. Finally, although extensive efforts were made to ensure the inclusion of all authors and their associated publications, we cannot ensure that some authors were missed and therefore, not included in our final statistical analysis. Similarly, our sample included one-quarter of dermatology residency programs listed on the Doximity website. The selection of a different time frame or medical specialty may yield varying results. In regard to strengths, data extraction was conducted in a duplicate and masked fashion, which is considered the gold standard by the Cochrane collaboration [37]. The second strength is the transparent and reproducible nature of our study. For ensuring transparency, our protocol was published in the Open Science Framework before commencing the study.

### Conclusions

Our results highlight research productivity before and during residency training as a potential marker for continued academic success in the field of dermatology. In addition, early scholarly involvement may be associated with successful matching into competitive subspecialty fellowships within the field of dermatology, as well as the pursuit of careers in academic medicine.

## Abbreviations

ACGME: Accreditation Council for Graduate Medical Education
PD: program director
USMLE: United States Medical Licensing Exam
